# Altered central and blood glutathione in Alzheimer’s disease and mild cognitive impairment: a meta-analysis

**DOI:** 10.1186/s13195-022-00961-5

**Published:** 2022-02-05

**Authors:** Jinghan Jenny Chen, Mathura Thiyagarajah, Jianmeng Song, Clara Chen, Nathan Herrmann, Damien Gallagher, Mark J. Rapoport, Sandra E. Black, Joel Ramirez, Ana C. Andreazza, Paul Oh, Susan Marzolini, Simon J. Graham, Krista L. Lanctôt

**Affiliations:** 1 Neuropsychopharmacology Research Group, Hurvitz Brain Sciences Program, Sunnybrook Research Institute, 2075 Bayview Avenue, Room FG52, Toronto, ON M4N 3M5 Canada; 2 Department of Pharmacology and Toxicology, University of Toronto, Toronto, ON Canada; 3 Department of Psychiatry, University of Toronto, Toronto, ON Canada; 4 Geriatric Psychiatry, Sunnybrook Health Sciences Centre, Toronto, ON Canada; 5 Evaluative Clinical Sciences, Hurvitz Brain Sciences Program, Sunnybrook Research Institute, Toronto, ON Canada; 6 KITE-Toronto Rehabilitation Institute, University Health Network, Toronto, ON Canada; 7 Physical Sciences, Hurvitz Brain Sciences Program, Sunnybrook Research Institute, Toronto, ON Canada

**Keywords:** Glutathione, Oxidative stress, Antioxidant, Alzheimer disease, Cognitive impairment, Biomarkers, Meta-analysis

## Abstract

**Background:**

Increasing evidence implicates oxidative stress (OS) in Alzheimer disease (AD) and mild cognitive impairment (MCI). Depletion of the brain antioxidant glutathione (GSH) may be important in OS-mediated neurodegeneration, though studies of post-mortem brain GSH changes in AD have been inconclusive. Recent in vivo measurements of the brain and blood GSH may shed light on GSH changes earlier in the disease.

**Aim:**

To quantitatively review in vivo GSH in AD and MCI compared to healthy controls (HC) using meta-analyses.

**Method:**

Studies with in vivo brain or blood GSH levels in MCI or AD with a HC group were identified using MEDLINE, PsychInfo, and Embase (1947–June 2020). Standardized mean differences (SMD) and 95% confidence intervals (CI) were calculated for outcomes using random effects models. Outcome measures included brain GSH (Meshcher-Garwood Point Resolved Spectroscopy (MEGA-PRESS) versus non-MEGA-PRESS) and blood GSH (intracellular versus extracellular) in AD and MCI. The *Q* statistic and Egger’s test were used to assess heterogeneity and risk of publication bias, respectively.

**Results:**

For brain GSH, 4 AD (AD=135, HC=223) and 4 MCI (MCI=213, HC=211) studies were included. For blood GSH, 26 AD (AD=1203, HC=1135) and 7 MCI (MCI=434, HC=408) studies were included. Brain GSH overall did not differ in AD or MCI compared to HC; however, the subgroup of studies using MEGA-PRESS reported lower brain GSH in AD (SMD [95%CI] −1.45 [−1.83, −1.06], *p*<0.001) and MCI (−1.15 [−1.71, −0.59], *z*=4.0, *p*<0.001). AD had lower intracellular and extracellular blood GSH overall (−0.87 [−1. 30, −0.44], *z*=3.96, *p*<0.001). In a subgroup analysis, intracellular GSH was lower in MCI (−0.66 [−1.11, −0.21], *p*=0.025). Heterogeneity was observed throughout (*I*^2^ >85%) and not fully accounted by subgroup analysis. Egger’s test indicated risk of publication bias.

**Conclusion:**

Blood intracellular GSH decrease is seen in MCI, while both intra- and extracellular decreases were seen in AD. Brain GSH is decreased in AD and MCI in subgroup analysis. Potential bias and heterogeneity suggest the need for measurement standardization and additional studies to explore sources of heterogeneity.

**Supplementary Information:**

The online version contains supplementary material available at 10.1186/s13195-022-00961-5.

## Background

 Alzheimer’s disease (AD) is the most common form of dementia representing up to 70% of all cases [1]. In AD, the brain shows hallmark features of amyloid beta (Aβ) plaque accumulation and neurofibrillary tangles formed by hyperphosphorylated tau protein [2], although prior to diagnosis, a series of neuropathological changes and cognitive decline occur [3]. Mild cognitive impairment (MCI), characterized by deficits beyond that anticipated for an individual’s age and education, but without functional impairment, is often the earliest clinical stage of AD [4]. Those with MCI have greater risk of conversion to AD than the normal population, with conversion rates ranging from 10 to 36% over a 2-year period depending on the methods used and the population under study [5].

Currently, there are no approved pharmacological treatments for MCI, although MCI is recognized to provide a window of opportunity to address modifiable risk factors and potentially prevent further progression to dementia [6]. For AD, approved interventions such as cholinesterase inhibitors and NMDA antagonists have modest effects on cognitive decline but are not able to reverse the course of disease [7]. Development of interventions targeting amyloid beta plaques and tau protein tangles also have not been successful [8, 9], and the number of phase 3 trials focused on amyloid intervention has decreased since 2019 [10]. Current phase 2 and 3 clinical trials have shifted focus to other interventions targeting tauopathy, synaptic plasticity, neuroprotection, and/or inflammation [11]. Overall, this suggests the need to identify additional mechanisms that may contribute to progression of AD.

Increasing evidence implicates oxidative stress (OS) with age-related neurodegeneration, neurotoxicity, and neuronal loss [12]. The brain is particularly susceptible to OS due to high metabolism required to maintain synaptic activity, and increased OS is associated with AD and MCI. Literature suggests antioxidant depletion and altered endogenous antioxidant systems precedes OS [12, 13]. Glutathione (GSH) is the primary antioxidant defense molecule in the brain [12]. It is a tripeptide of glutamate, cysteine, and glycine and exerts antioxidant effects through donating a reducing equivalent to a reactive oxygen species to neutralize it [14]. This reaction can occur both non-enzymatically and through catalysis by glutathione peroxidase [14, 15]. In vitro and animal studies suggests that GSH depletion plays an important role in OS-mediated neuronal death and is implicated in neuronal loss in several neurodegenerative diseases, such as Parkinson’s disease [16], AD [17], and amyotrophic lateral sclerosis [12], making it a potential therapeutic target to prevent or reduce neurodegeneration.

A previous meta-analysis of GSH levels in post-mortem AD brain tissue found no evidence of significant change in GSH in AD compared to controls across several brain regions [18]. The authors also noted that little quantitative post-mortem data were available for MCI. However, the quality of post-mortem data can be variable, as GSH concentration in the brain drops rapidly after death and is affected by many pre- and post-mortem factors [19, 20]. Recent studies have measured in vivo GSH in the brain using magnetic resonance spectroscopy (MRS) and peripherally in the blood [21–23]. These in vivo measures are arguably more accurate and provide additional information to help determine if GSH may be considered a therapeutic target.

Therefore, the focus of the present work is to review quantitatively the in vivo GSH changes in the brain and the periphery in AD and MCI compared to controls, using meta-analytic methods.

## Methods

### Data sources

The methodology outlined by the PRISMA guidelines was used for this review [24]. Articles published before June 2020 were searched using MEDLINE, PsychInfo, Embase, and CINAHL databases for original reports containing in vivo brain or blood measurements of GSH in MCI and/or AD patients and healthy controls. A sample search strategy of brain GSH (for Embase) is detailed in Table 1.

**Table 1:** Sample search strategy for Embase

Search strategy	
Population	“Alzheimer Disease” OR “Dementia” OR “Dementia, Vascular” OR “Dementia, multi-infarct” OR “cognitive dysfunction”
Method of measurement	“Magnetic resonance spectroscopy” OR “Proton Magnetic Resonance Spectroscopy”
Comparison	Mild Cognitively Impaired and/or Alzheimer Disease vs. Controls
Outcomes	“Glutathione” OR “Oxidative Stress” OR “Antioxidants”
Type of question	Screening/diagnosis/prognosis
Type of study	Randomized controlled trials, controlled trials, prospective/cohort/longitudinal follow-up studies, cross sectional studies, case control studies EXCLUDE: case reports, research in progress, conference abstracts, dissertations, books, scientific meeting reports

### Study selection

Two of the 3 independent reviewers (JC, MT, and JS) assessed each retrieved reference. Screening was done by reviewing reference abstracts to exclude case reports, research in progress, conference abstracts, dissertations, books, and scientific meeting reports. Full-text articles were then assessed. Study inclusion criteria were (1) original clinical studies reporting in vivo GSH levels in the brain, serum, plasma, or blood cells (2); clinical diagnosis of MCI or AD using standardized diagnosis; and (3) inclusion of a medically healthy and cognitively intact control group. Studies measuring post-mortem GSH concentrations without any measures of in vivo GSH were excluded. At least 2 reviewers examined each article for inclusion eligibility independently, results were compared and disagreements regarding inclusion were reached by consensus.

### Data extraction

Mean (±SD) GSH concentrations for MCI, AD, and control groups were extracted from each article. Study and participant characteristics were collected using a standardized form. Population characteristics (mean age, sex proportion, years of education, cognitive test scores) and study variables (inclusion criteria, diagnosis method, GSH measurement methodology) were also extracted where available. Reporting the quality and risk of bias items were evaluated by at least 2 raters using items from the Newcastle Ottawa Scale and the Cochrane Collaboration’s risk of bias assessment tool as done previously [25]. Corresponding authors of publications were contacted for missing data. When studies reported multiple brain regions or several blood components (plasma, serum, blood cells), each region or component was extracted as a sub-study. When possible, peripheral GSH measurements were converted to μM, μMol/gHb, or μMol/g protein as appropriate.

### Statistical analysis

StataIC 16 was used for the main and subgroup analyses. Standardized mean differences (SMD) and 95% confidence intervals were calculated for each outcome using a random effects model [24]. As studies used different scales of measurement, SMDs were chosen to summarize between group differences since it can better adjust for the different scaling used [26]. Random effects models are preferable when significant heterogeneity is expected because they account for variable underlying effects in estimates of uncertainty, including both within-study and between-study variances [27]. In brain GSH measurements, different acquisition methodologies, internal references, and brain regions have been used. In blood GSH, different assays and blood components were also used. These factors were expected to contribute to significant heterogeneity.

The *Q* statistic was calculated using a chi-square analysis to assess heterogeneity among combined results. A significant *Q* statistic indicates diversity in the characteristics of the combined results. Inconsistency was calculated using an *I*^2^ statistic to determine the impact of heterogeneity. The risk of publication bias was assessed quantitatively with the Egger’s test [28].

Potential heterogeneity was explored with inverse-variance weighted meta-regression analyses and subgroup analysis. Meta-regression regressed the standard mean differences against mean age, sex proportion, or mean Mini-Mental State Examination (MMSE) scores if at least 10 independent studies were included based on Cochrane recommendations. Subgroups were determined a priori to determine if MRS acquisition protocol, internal reference, or brain regions contributed to heterogeneity in brain GSH measurements. In the blood, subgroup analysis was performed to determine if intracellular (erythrocytes and whole blood) or extracellular (plasma and serum) components contributed to heterogeneity in blood GSH, as well as the assay used to measure GSH, namely assays using 5,5′-dithio-bis(2-nitrobenzoic acid) (DTBN) and o-phthalaldehyde (OPA).

## Results

### Literature search

#### Brain GSH literature findings

**Fig. 1:**
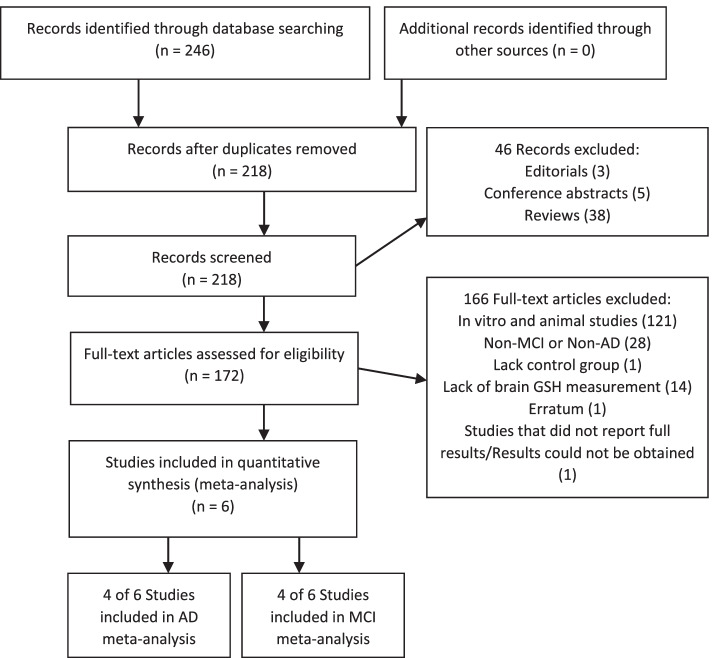
Search and selection of articles relevant to brain GSH in AD and MCI

The search returned 218 unique records (Fig. 1). Of the records screened, 46 studies were excluded as they were non-clinical studies (including reviews, editorials, and or conference abstracts), 121 studies were excluded because those studies involved non-human subjects, 28 studies were excluded as they were not conducted in AD or MCI patients, 1 study was excluded as it did not have a healthy control group, and 14 studies were excluded as they did not measure GSH in the brain. One paper was excluded as it was an erratum clarification that was not relevant to the results. One additional study was excluded from quantitative analysis as full results could not be obtained. A total of 4 studies were included in the AD brain GSH meta-analysis [29–32], and 4 studies were included in the MCI analysis [30, 33–35] (Table 2). Studies reporting multiple brain locations were analyzed as sub-studies, and when bilateral measures were available, the left and right voxels were averaged. A total of 7 studies and sub-studies were included for AD brain GSH analysis and 8 studies and sub-studies were included for MCI analysis. Assessment of included studies showed a consistently low risk of bias in the brain GSH literature (Table 3).

**Table 2:** Summary of included studies

First author, publication year	Tissue	Analysis method	*N* (case/HC)	MMSE of case	Mean age	% Male
Brain GSH studies—AD						
*Mandal, 2015* [35]	Brain: hippocampus, frontal cortex	MEGA-PRESS, reference: water	19/28	23.6	66.2	68%
*Marjanska, 2019* [32]	Brain: posterior cingulate, occipital cortex	STEAM, reference: water	16/33	19	73.2	82%
*Mullins, 2018* [31]	Brain: posteromedial cortex	J-PRESS, reference: creatine	27/54	25.4	72.2	57%
*Shukla, 2019*	Brain: anterior and posterior cingulate	MEGA-PRESS, reference: water	18/27	N/A	69.4	70%
Brain GSH studies—MCI						
*Duffy, 2014* [33]	Brain: anterior and posterior cingulate	PRESS, reference: creatine	54/41	28.7	68	52%
*Mandal, 2015* [35]	Brain: hippocampus, frontal cortex	MEGA-PRESS, reference: water	22/28	24.2	66	65%
*Oeltzschner, 2019* [34]	Brain: anterior and posterior cingulate	STEAM, reference: creatine	13/26	28.1	69	65%
*Shukla, 2019*	Brain: anterior and posterior cingulate	MEGA-PRESS, reference: water	19/28	N/A	66.6	71%
Blood GSH studies—AD						
*Arslan, 2016* [36]	Erythrocyte	DTNB	24/15	N/A	73.5	77%
*Aybek, 2007* [37]	Serum	DTNB	62/56	17.8	72.1	47%
*Bai, 2018* [38]	Plasma	DTNB	16/16	13.1	N/A	N/A
*Baldeiras, 2008* [39]	Plasma and erythrocyte	OPA	42/37	20.9	70.8	39%
*Bermejo, 2008* [40]	Erythrocyte	OPA	45/28	N/A	80.0	N/A
*Bicikova, 2004* [41]	Serum	HPLC	21/40	N/A	72.5	44%
*Fernandes, 1999* [45]	Plasma and erythrocyte	OPA	74/35	N/A	67.2	45%
*Gironi, 2011* [43]	Serum	HPLC	25/66	18.9	72.5	36%
*Gironi, 2014* [42]	Erythrocyte	HPLC	37/28	N/A	76.1	40%
*Gubandru, 2013* [44]	Plasma	DTNB	21/10	10.51	79.9	52%
*Hernanz, 2007* [46]	Plasma	HPLC	25/44	N/A	73.4	52%
*Kliumiuk, 2019* [47]	Plasma	DTNB	15/50	13.4	80.9	30%
*Kosenko, 2016* [48]	Erythrocyte	DTNB	12/14	N/A	76.1	35%
*Krishnan, 2014* [49]	Plasma and erythrocyte	DTNB	30/40	4	66.3	54%
*Kurup, 2003* [50]	Erythrocyte	DTNB	15/15	N/A	N/A	N/A
*Liu, 2005* [51]	Plasma,erythrocyte, and leukocyte	HPLC	33/20	17.7	75.9	45%
*Martinez de Toda, 2019* [52]	Whole blood	OPA	20/30	N/A		55%
*McCaddon, 2003* [53]	Plasma	HPLC	50/57	18	79.0	37%
*Mohamed, 2019* [54]	Serum	ELISA	50/25	19.2	69.8	50%
*Prendecki, 2018* [55]	Plasma	HPLC	88/80	15.3	73.9	73%
*Puertas, 2012* [56]	Plasma	DTNB	46/46	22	74.2	39%
*Rani, 2017* [57]	Plasma	DTNB	45/45	3.5	69.6	N/A
*Riveron, 2007* [58]	Plasma	DTNB	25/30	N/A	N/A	N/A
*Sadhu, 2014* [59]	Plasma	DTNB	104/93	6.4	N/A	54%
*Tabet, 2002* [60]	Plasma	Commercial Assay Kit	31/30	13.9	N/A	46%
*Vida, 2018* [61]	Whole blood, neutrophil, lymphocyte	OPA	44/38	19.3	75.9	41%
Blood GSH studies—MCI						
*Baldeiras, 2008* [39]	Plasma and erythrocyte	OPA	85/37	27	70.3	39%
*Bermejo, 2008* [40]	Erythrocyte	OPA	34/28	27	78.3	N/A
*Gironi, 2011* [43]	Serum	HPLC	20/66	N/A	71.4	33%
*Gironi, 2014* [42]	Erythrocyte	HPLC	26/28	21.5	76.5	43%
*Hernanz, 2007* [46]	Plasma	HPLC	26/44	N/A	74.4	51%
*Martinez de Toda, 2019* [52]	Whole blood	OPA	20/30	25	N/A	50%
*Yuan, 2016* [62]	Plasma	Commercial assay kit	138/138	N/A	64.5	51%

Abbreviations: *AD* Alzheimer disease, *DSM* Diagnostic And Statistical Manual of Mental Disorders, *DTBN* 5,5′-dithio-bis(2-nitrobenzoic acid), *ELISA* enzyme-linked immunosorbent assay, *GSH* glutathione, *HC* healthy control, *HPLC* high performance liquid chromatography, *MCI* mild cognitive impairment, *MEGA-PRESS* Meshcher-Garwood Point-Resolved Spectroscopy, *MMSE* Mini-Mental State Examination, *N/A* not available, *OPA* O-Phthalaldehyde, *PRESS* Point-Resolved Spectroscopy, *SMD* standardized mean difference, *STEAM* STimulated Echo Acquisition Mode

**Table 3:** Study quality and risk of bias assessment. Studies were assessed using items from the Newcastle Ottawa Scale and the Cochrane Collaboration’s risk of bias assessment tool, addressing key methodological criteria relevant to included studies. (+ indicates yes; −, no; ?, uncertain)

		Demographics reported	Medical comorbidities reported	Excluded medical comorbidities	Non-retrospective design	Standardized criteria used for diagnosis	Reported medication use	Excluded use of antioxidants	Representative population
First author	Year	General risk of bias items							
**Brain GSH**									
*Duffy*	2014	+	-	+	+	+	+	-	+
*Mandal*	2015	+	-	+	+	+	-	-	+
*Marjanska*	2019	+	-	+	+	+	-	-	+
*Mullins*	2018	+	-	+	+	+	-	-	?
*Oeltzschner*	2019	+	-	+	+	+	-	-	+
*Shukla*	2019	+	-	+	+	+	-	-	+
**Blood GSH**									
*Arslan*	2016	+	-	+	+	+	-	-	+
*Aybek*	2007	+	-	-	+	+	-	-	+
*Bai*	2018	-	-	+	+	+	-	-	+
*Baldeiras*	2008	+	?	+	+	+	+	+	+
*Bermejo*	2008	?	-	+	+	+	-	-	?
*Bicikova*	2004	+	+	+	+	+	-	-	+
*Gironi*	2011	+	-	+	+	+	+	+	+
*Gironi*	2014	+	-	+	+	+	-	-	+
*Gubandru*	2013	+	+	+	+	?	+	+	?
*Fernandes*	1999	+	-	+	?	+	-	-	+
*Hernanz*	2007	+	-	-	+	+	-	-	+
*Klimiuk*	2019	+	+	+	+	?	-	+	+
*Kosenko*	2016	+	-	-	+	+	-	-	+
*Krishnan*	2014	+	-	+	+	+	-	-	+
*Kurup*	2003	-	+	-	+	+	?	-	?
*Liu*	2005	+	-	+	+	+	-	-	+
*McCaddon*	2003	+	+	+	+	+	-	-	+
*Martinez de Toda*	2019	+	-	+	+	+	-	-	+
*Mohamed*	2019	+	+	+	+	+	+	-	+
*Prendecki*	2018	+	-	-	+	+	+	-	+
*Puertas*	2012	+	-	+	+	+	-	+	+
*Rani*	2017	+	-	+	+	+	-	-	+
*Riveron*	2007	-	-	+	+	+	-	+	+
*Sadhu*	2014	-	+	+	+	+	?	-	?
*Tabet*	2002	+	-	-	+	+	-	-	+
*Vida*	2018	+	-	+	+	+	-	-	+
*Yuan*	2016	+	-	+	+	+	-	+	+

		Representative population	Cognitively intact control group	Community controls	Similarly aged controls	Similar gender proportions in controls	Similar in other characteristics	Assessed for cognitive impairment	Likelihood of high overall quality
First author	Year	Control items							
**Brain GSH**									
*Duffy*	2014	+	+	+	+	+	+	+	+
*Mandal*	2015	+	+	+	+	+	+	+	+
*Marjanska*	2019	+	+	?	+	-	?	+	+
*Mullins*	2018	?	?	?	+	+	?	-	-
*Oeltzschner*	2019	+	+	+	-	-	?	+	+
*Shukla*	2019	?	?	+	+	-	?	-	-
**Blood GSH**									
*Arslan*	2016	?	+	-	+	-	+	+	-
*Aybek*	2007	?	+	-	+	+	?	-	-
*Bai*	2018	+	+	-	?	?	?	-	-
*Baldeiras*	2008	+	+	?	+	-	+	+	+
*Bermejo*	2008	?	+	-	+	?	?	-	-
*Bicikova*	2004	+	+	?	+	+	+	+	+
*Gironi*	2011	+	+	+	-	+	?	+	+
*Gironi*	2014	+	+	-	+	-	?	+	+
*Gubandru*	2013	+	?	?	+	+	?	-	+
*Fernandes*	1999	+	+	?	-	+	?	+	-
*Hernanz*	2007	+	+	?	+	+	+	+	+
*Klimiuk*	2019	+	+	-	+	+	+	+	+
*Kosenko*	2016	+	?	?	+	-	+	-	-
*Krishnan*	2014	+	+	?	+	+	+	+	+
*Kurup*	2003	+	?	+	?	?	?	-	-
*Liu*	2005	+	+	+	+	+	?	+	+
*McCaddon*	2003	+	+	+	+	+	+	+	+
*Martinez de Toda*	2019	+	+	-	+	+	+	+	+
*Mohamed*	2019	+	+	?	+	+	+	+	+
*Prendecki*	2018	-	+	?	+	+	?	+	+
*Puertas*	2012	?	+	-	+	+	?	-	-
*Rani*	2017	+	+	?	+	+	?	+	+
*Riveron*	2007	+	?	?	?	?	?	?	-
*Sadhu*	2014	?	?	?	?	?	?	+	-
*Tabet*	2002	+	+	+	-	-	?	+	-
*Vida*	2018	?	+	-	+	-	?	+	-
*Yuan*	2016	+	?	+	+	+	+	-	+

#### Blood GSH literature findings

**Fig. 2:**
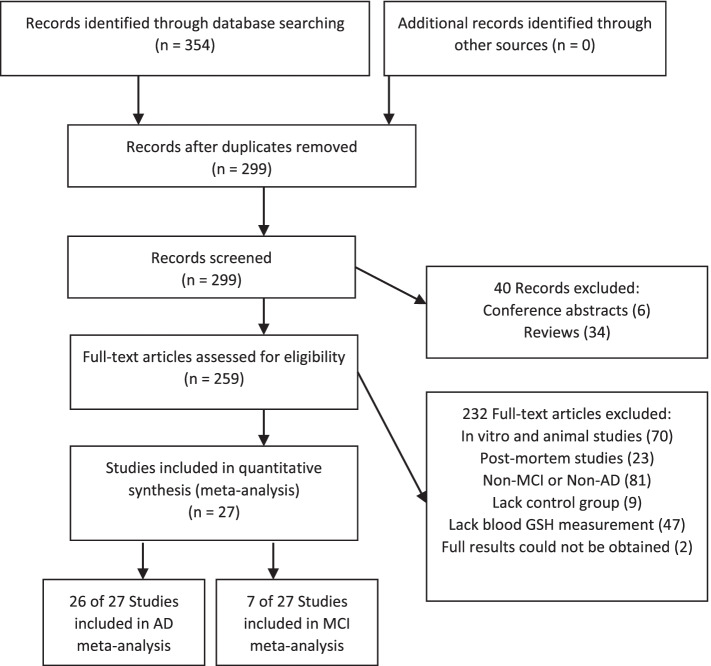
Search and selection of articles relevant to blood GSH in AD and MCI

The search returned 299 unique records (Fig. 2). Of the records screened, 40 studies were excluded as they were non-clinical studies (including reviews, editorials, and or conference abstracts); 70 studies were excluded because these studies involved non-human subjects; 81 studies were excluded as they were not conducted in AD or MCI patients; 23 studies were excluded as they were post-mortem studies; 9 studies were excluded as they did not include a healthy control group; 47 studies were excluded as they did not measure GSH in whole blood, plasma, or serum; and 2 were excluded as full results could not be obtained. A total of 27 studies qualified, with 26 of these studies being included in the AD blood GSH meta-analysis [36–61], and 7 of these studies being included in the MCI analysis [39, 40, 42, 43, 46, 52, 62]. Studies reporting GSH levels in different blood components (plasma, serum, blood cells) were analyzed as sub-studies, with a total of 33 studies/sub-studies used for AD blood GSH analysis and 8 studies/sub-studies used for MCI blood GSH analysis. The risk of bias was variable in the AD blood GSH literature but consistently low in MCI blood GSH literature (Table 3).

#### Diagnostic criteria used in AD and MCI

AD patients were identified primarily using the Diagnostic and Statistical Manual of Mental Disorders (DSM) [63] and/or the National Institute of Neurological and Communicative Diseases and Stroke/Alzheimer’s Disease and Related Disorders Association [64]. The National Institute on Aging-Alzheimer’s Association diagnostic guidelines [65], The Dementia Rating Scale-2 [66], International Classification of Diseases 10th Revision [67], and the Consortium to Establish a Registry for Alzheimer’s Disease [68] neuropsychological battery were used in 5 GSH studies, respectively [31, 37, 48, 50, 59]. In studies examining blood GSH in AD, 7 studies used the Hachiniski Ischaemic Score (HIS ≤ 4) to differentiate those with AD from those with potential vascular causes [41, 46, 55, 58, 60, 61, 69], and 5 of those studies further used neuroimaging to support diagnosis [55, 58, 60, 61, 69] (Table 2).

For MCI patient samples, the Petersen criteria [70] were commonly used to diagnose MCI, though other studies used revised Petersen criteria [71], or a combination of the Montreal Cognitive Assessment, DSM-IV, Clinical Dementia Rating, and Mini-Mental State Examination [34, 62]. While amnestic-type MCI patients were specifically selected in 2 blood GSH studies [42, 43], most of the studies measuring blood GSH and all the studies measuring brain GSH either did not specify or included both amnestic and non-amnestic patients (Table 2).

#### Brain GSH concentrations and investigating heterogeneity

**Fig. 3:**
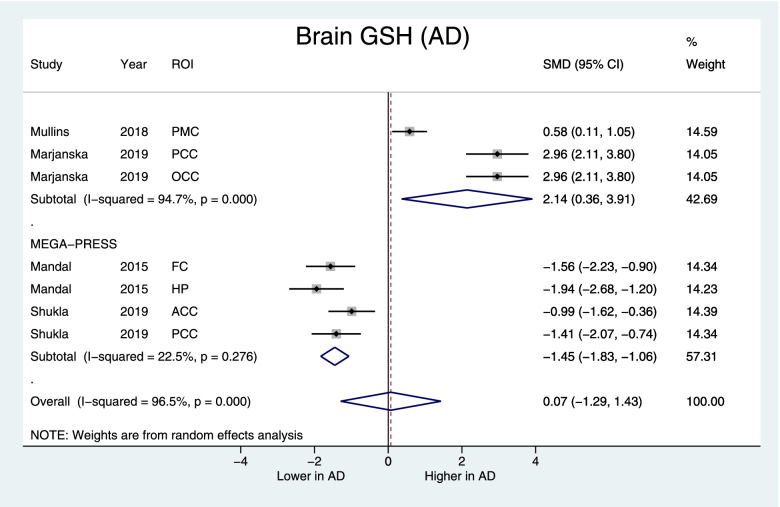
Forest plot displaying brain GSH concentrations in AD and control subjects, with the subgroup of studies using the MEGA-PRESS protocol at the bottom. Shown are the standardized mean differences (SMD) and 95% confidence intervals (95% CI). Negative values denote lower GSH in AD subjects while positive values denote higher in GSH in AD compared to controls. Pooled SMD [95% CI] = −0.07 [−1.29, 1.43], *z*=0.1, *p*=0.92, MEGA-PRESS subgroup: SMD [95% CI] = −1.45 [−1.83, −1.06], *z*=7.41, *p*<0.001. ROI indicates the region of interest: PMC posteromedial cortex, PCC posterior cingulate cortex, OCC occipital cortex, HP hippocampus, FC frontal cortex, ACC anterior cingulate cortex

**Fig. 4:**
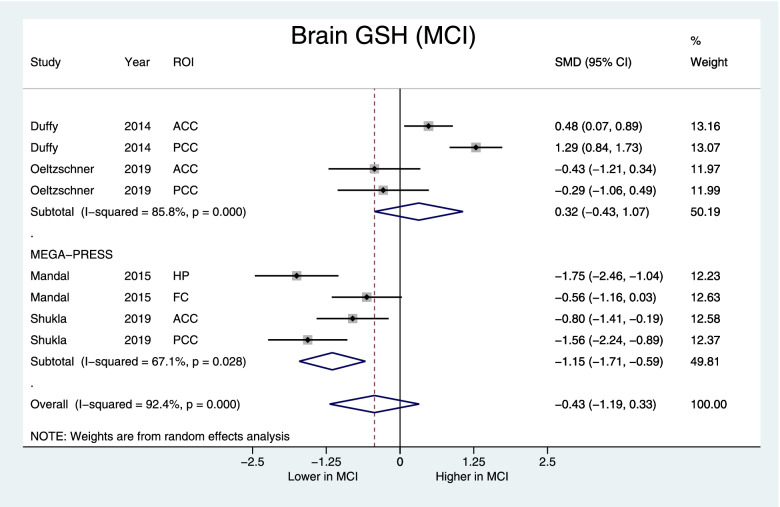
Forest plot displaying brain GSH concentrations in MCI and control subjects, with the subgroup of studies using the MEGA-PRESS protocol at the bottom. Shown are the standardized mean differences (SMD) and 95% confidence intervals (95% CI). Negative values denote lower GSH in MCI subjects while positive values denote higher in GSH in MCI compared to controls. Pooled SMD [95% CI] = −0.43 [−1.19, 0.33], *z*=1.12, *p*=0.26, MEGA-PRESS subgroup: SMD [95% CI] = −1.15 [−1.71, −0.59], *z*=4.0, *p*<0.001. ROI indicates the region of interest: ACC anterior cingulate cortex, PCC posterior cingulate cortex, FC frontal cortex, HP hippocampus

Brain GSH did not differ in AD (pooled SMD [95%CI] = 0.07 [−1.29, 1.43], *p*=0.6) and MCI (pooled SMD [95%CI] = −0.43 [−1.19, 0.33], *p*=0.26) compared to healthy controls. Significant heterogeneity was found in both AD (*I*^2^=96.5%, *p*<0.001) and MCI (*I*^2^=92.4%, *p*<0.001) and supported the use of random effect models. Subgroup analysis evaluating the use of MRS acquisition methods found that Meshcher-Garwood Point Resolved Spectroscopy (MEGA-PRESS) studies had reduced heterogeneity (AD: *I*^2^=22.5%, *p*=0.28, MCI: *I*^2^=67.1%, *p*=0.03), and non-MEGA-PRESS studies remained heterogeneous (*I*^2^=94.7%, *p*<0.001). In the MEGA-PRESS subgroup, brain GSH was lower in both AD (SMD [95%CI] = −1.45 [−1.83, −1.06], *z*=7.41, *p*<0.001) (Fig. 3) and MCI (−1.15 [−1.71, −0.59], *z*=4.0, *p*<0.001) groups (Fig. 4). Subgroup analyses of different brain regions and use of creatine or water as the reference molecule did not significantly reduce heterogeneity in brain GSH measurements (data not shown), with the exception of the study by Marjanska et al. 2019, use of water as a reference molecule overlapped with MEGA-PRESS studies in AD and MCI (Table 1).

#### Blood GSH concentrations and investigating heterogeneity

**Fig. 5:**
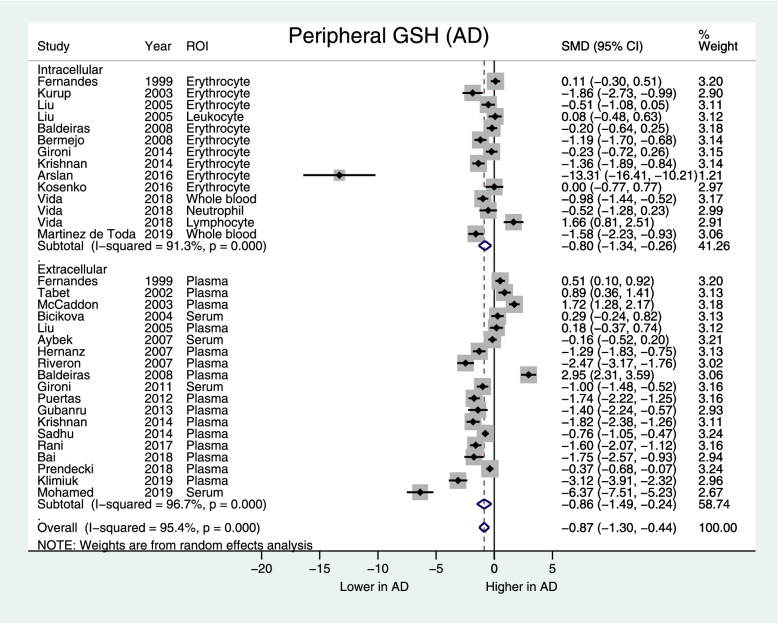
Forest plot displaying blood GSH concentrations in AD and control subjects, by the intracellular and extracellular GSH subgroups. Shown are the standardized mean differences (SMD) and 95% confidence intervals (95% CI). Pooled SMD [95% CI] = −0.87 [−1.30, −0.44], *z*=3.96, *p*<0.001. Positive values denote higher in GSH in AD while negative values denote higher GSH in control subjects. ROI region of interest

**Fig. 6:**
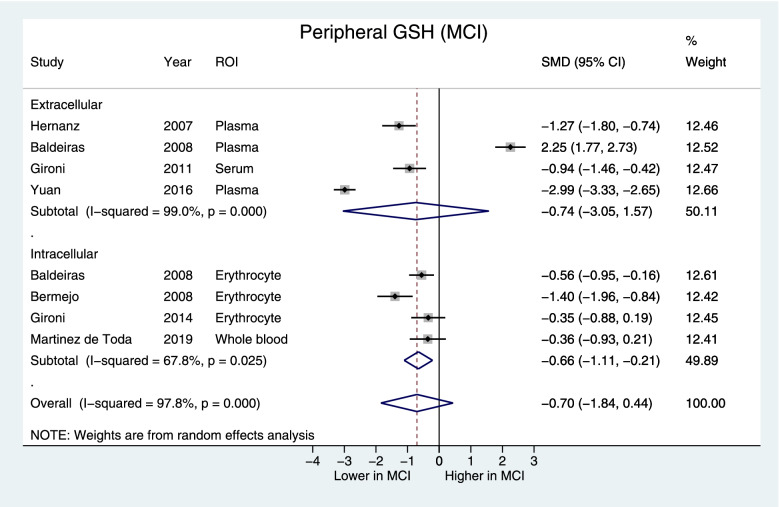
Forest plot displaying blood GSH concentrations in MCI and control subjects by the intracellular and extracellular GSH subgroups. Shown are the standardized mean differences (SMD) and 95% confidence intervals (95% CI). Pooled SMD [95% CI] = −0.70 [−1.84, 0.44], *z*=1.12, *p*=0.23, the intracellular subgroup SMD [95% CI] = −0.66 [−1.11, −0.21], *z*=4.0, *p*=0.004. Positive values denote higher in GSH in MCI while negative values denote higher GSH in control subjects. ROI region of interest

**Fig. 7:**
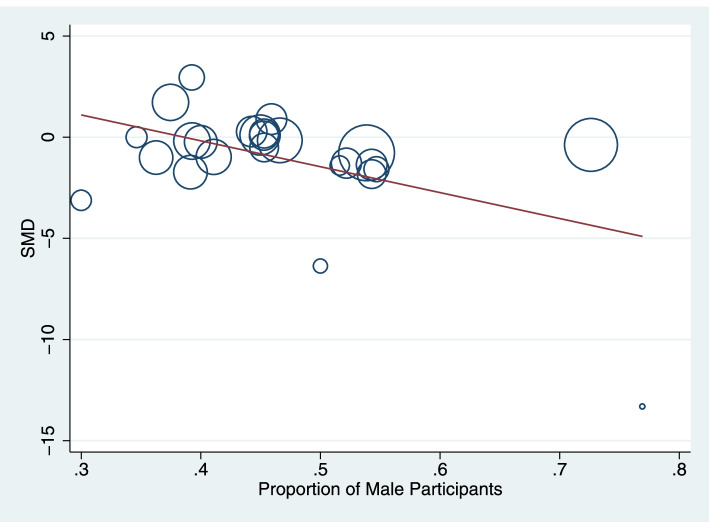
Meta-regression demonstrating inverse correlation between the proportion of male study participants and the standardized mean difference (SMD) of blood GSH level between AD participants and healthy controls. The size of the circles is proportional to study weights

Blood GSH was lower in AD (SMD [95%CI] = −0.87 [−1.30, −0.44], *z*=3.96, *p*<0.001) but not in MCI groups compared to controls (SMD [95%CI] = −0.70 [−1.84, 0.44], *z*=1.12, *p*=0.23). Significant heterogeneity was observed for both analyses (AD: *I*^2^=95.4%, *p*<0.001, MCI: *I*^2^=97.8%, *p*<0.001). In AD, both intracellular and extracellular blood GSH were lower (intracellular SMD [95%CI] = −0.80 [−1.34, −0.26], *p*=0.004; extracellular SMD [95%CI] = −0.86 [−1.49, −0.24], *p*=0.007) without reduced heterogeneity (AD intracellular: *I*^2^=91.3%, *p*<0.001; extracellular: *I*^2^=96.7%, *p*<0.001) (Fig. 5). Intracellular GSH was lower in MCI (SMD [95%CI] = −0.66 [−1.11, −0.21], *p*=0.025) with reduced but still significant heterogeneity (MCI intracellular: *I*^2^=67.8%, *p*<0.025) (Fig. 6). Subgroup analysis of GSH assay type did not significantly reduce heterogeneity in blood GSH measurements. Meta-regression showed that studies having a higher proportion of male participants reported greater decreases in GSH levels in AD compared to controls (*p* = 0.01, *I*^2^_res_ = 95.83%, *R*_adj_^2^ = 18.9%) (Fig. 7). Meta-regressions with the mean age and MMSE scores did not significantly reduce heterogeneity (data not shown).

#### Effect of study bias, publication bias, and small-study effects

In all analyses, the pooled estimated SMDs for the subgroups of studies deemed to have low bias was within the 95% CI of the overall (Table 4), suggesting the impact of studies with higher bias was small. Publication bias was not detected by funnel plots, Egger’s, or trim and fill tests in AD brain GSH literature and MCI blood GSH literature. However, Egger’s test detected a significant risk of publication bias in MCI brain GSH literature (bias [95%CI] = −11.28 [−20.6, −1.95], *p*=0.03) and AD blood GSH literature (bias [95%CI] = −5.18 [−9.96, −0.40], *p*=0.035). Blood GSH remained lower in AD compared to controls after adjusting for potential publication bias using trim and fill (estimated SMD [95%CI] = −0.87 [−1.30, −0.44], *p*<0.001).

**Table 4:** Summary of outcomes for peripheral brain and blood GSH by qualitative assessment. Studies with 10 or more items rated as “yes” in the qualitative risk of bias assessment were categorized as likely to be “low risk of bias”

Studies and sub-studies (*n*)			*SMD [95% CI]*	*z*	*p*	*X*^*2*^	*I*^*2*^ *(%)*	*p*
Brain GSH	AD (all)	(7)	0.07 [-1.29, 1.43]	0.1	0.92	173.46	96.5	<0.001
	AD (low risk of bias)	(4)	0.59 [-2.04, 3.23]	0.44	0.66	141.01	97.9	<0.001
	MCI (all)	(8)	-0.43 [-1.19, 0.33]	1.12	0.26	92.32	92.4	<0.001
	MCI (low risk of bias)	(6)	-0.19 [-1.04, 0.67]	0.43	0.67	64.08	92.2	<0.001
Blood GSH	AD (all)	(31)	-1.18 [-1.65, -0.71]	4.9	<0.001	728.87	95.9	<0.001
	AD (low risk of bias)	(17)	-0.87 [-1.56, -0.20]	2.53	0.01	447.49	96.4	<0.001
	MCI (all)	(8)	-0.70 [-1.84, 0.44]	1.21	0.23	322.88	97.8	<0.001
	MCI (low risk of bias)	(6)	-0.65 [-2.13, 0.84]	0.85	0.39	315.61	98.4	<0.001

## Discussion

### Brain GSH concentrations

This meta-analysis did not find significant differences between MCI and controls, nor AD vs. controls in in vivo brain GSH overall; however, subgroup analysis suggests that brain GSH may be decreased in AD and MCI in studies using MEGA-PRESS to acquire GSH measurements. GSH is an essential antioxidant in brain cells that detoxifies reactive oxygen species, and in vitro studies have linked GSH homeostasis disruption to oxidative stress in neurological diseases [14, 72]. Increased lipid peroxidation and oxidative stress have been described in AD and MCI [73–75]; however, brain GSH has not been as well-characterized. The results of this in vivo brain GSH study mirrors a previous meta-analysis examining post-mortem GSH levels in brain tissue, where they reported that in post-mortem AD brain samples, GSH appeared to be unchanged across several brain regions [18]. It should be noted that GSH data obtained from post-mortem brain samples are variable in quality, as brain GSH is affected by many pre- and post-mortem factors and changes quickly after death [19, 20].

Interestingly, the subgroup analysis of brain GSH suggested that studies using MEGA-PRESS to acquire brain GSH measurements reported lower brain GSH in both AD and MCI patients compared to controls. MEGA-PRESS, a modified PRESS sequence, is a standard technique used in MRS measurements of γ-aminobutyric acid [23] and has been adapted to measure GSH in normal subjects [21, 76] as well as in several patient populations such as schizophrenia [77, 78], Parkinson’s disease [16, 79], and pediatric populations [80]. Studies involving “phantom” test materials suggest that PRESS-acquired GSH may include oxidized GSH (GSSG) and that GSH edited MEGA-PRESS measurements give more precise values at lower GSH concentrations. The existing MRS studies measuring in vivo brain GSH in AD and MCI used several protocols, including STEAM [32, 34], PRESS [33], MEGA-PRESS [30, 35], and J-PRESS [31]. The high heterogeneity and significant risk of bias seen in these in vivo brain GSH studies suggests the need to standardize in vivo GSH measurement methodology. And while qualitative assessment of brain GSH studies is relatively consistent, there may be other factors contributing to heterogeneity. MEGA-PRESS may be a promising protocol, although the current MEGA-PRESS studies reporting brain GSH in MCI and AD were from a single research group, which may have artificially reduced heterogeneity.

Currently, in vivo brain markers in AD and MCI mainly include positron emission tomography scanning of amyloid, tau, and glucose metabolism, as well as brain structural imaging using MRI such as hippocampal atrophy [81]. However, it is now recognized that development and progression of AD is likely due to multiple etiologies, and there is increasing evidence implicating oxidative stress (OS) as an early event in the trajectory of MCI and AD [2, 18, 75]. Thus, examining in vivo brain GSH as a biomarker would complement the current arsenal of brain biomarkers and may aid in identifying and characterizing changes in the early stage of cognitive impairment or those who are at risk.

### Blood GSH concentrations

This meta-analysis found that in AD, there was a significant decrease in blood GSH compared to controls, but no difference between MCI and controls. Blood GSH measurements came from extracellular sources in serum and plasma, or intracellular sources in erythrocytes, whole blood (both erythrocytes and leukocytes), or leukocytes. In serum and plasma, reduced GSH is primarily released by hepatocytes for uptake by the kidney, lung, intestine, and other organs [14]. Therefore, in the periphery, extracellular GSH reflects the antioxidant capacity of the liver, and the liver, due to its function in metabolizing xenobiotics and endogenous molecules, has high antioxidant capacity [16]. In the intracellular compartment, erythrocytes perform de novo GSH synthesis [82], GSH is also important in activation of lymphocytes and regulation of immune response [61, 83, 84]. Thus, intracellular GSH may be more sensitive to early changes in GSH homeostasis than extracellular GSH. Indeed, in our subgroup analysis, intracellular blood GSH is decreased in MCI vs. controls, while both intra- and extracellular blood GSH is lowered in AD compared to controls. In our sample, 2 studies in AD [51, 61] specifically reported leukocyte GSH levels with none in MCI. In the context of literature suggesting that sustained immune response and elevation of proinflammatory cytokines in AD pathology [61, 85], additional studies to examine GSH changes in immune cells would be an important future direction. Nonetheless, our peripheral GSH findings suggests that intracellular GSH may be more sensitive to early stages of disease and that extracellular changes become apparent in more severe stages of cognitive impairment such as AD.

A variety of assays were used to measure serum, plasma, and intracellular GSH in MCI and AD populations, including assays using DTBN [36–38, 44, 48–50, 56–59], OPA [39, 40, 45, 52, 61], high performance liquid chromatography [42, 43, 46, 51, 53, 55], enzyme-linked immunosorbent assays [69], and other commercial assay kits [60, 62]. Although subgroup analyses found significant heterogeneity regardless of assay type, literature suggests that different assays have specific characteristics and potential pitfalls [82]. OPA-based assays are more sensitive but unstable, which affects accuracy and precision [86], whereas DTNB-based assays allow for determination of biothiols in the presence of other amino acids and polyphenolic antioxidants but are less sensitive [82]. Indeed, the high heterogeneity observed in the present study also corroborates the wide variation of GSH concentrations across different studies and laboratories.

### Population-based sources of heterogeneity

Other potential sources of heterogeneity may be related to the populations included in the studies. Sex differences in GSH and enzymes involved in its metabolism have been reported in healthy individuals [87], patients with AD [51], infants [88], and several animal models [89–91]. Higher antioxidant defense is seen in females and has been attributed to the ability of estrogen to upregulate expression of antioxidant enzymes [92]. In our analysis of blood GSH studies conducted in AD participants, the proportion of males significantly contributed to heterogeneity. Studies having higher proportion of male participants had larger SMDs, suggesting that AD studies with more male participants reported lower GSH compared to controls. Unfortunately, neither blood nor brain GSH publications in MCI were sufficiently numerous to support similar meta-regressions, but sexual dimorphism in GSH metabolism would be an important covariate to consider in future studies. Another potential source of heterogeneity is the presence of vascular disease in these samples. In studies examining blood GSH in AD, most studies did not examine potential vascular contributions. However, in studies examining brain GSH in AD, most studies excluded those with a history of stroke or transient ischemic attack. Oxidative stress has been identified as having an important role in cerebrovascular disease and given increasing recognition of the overlap between vascular dementia and AD (“mixed dementia”) and the contribution of vascular changes to AD [93] investigating potential effects of cerebrovascular disease as a covariate would be an important direction for future studies. There were also differences in the MCI populations included with most studies including unknown proportions of amnestic and non-amnestic patients. Amnestic MCI is associated with a higher risk of conversion to AD [94], but those with non-amnestic MCI are a heterogenous group with a higher risk of conversion to other dementias [95]. The MCI patients included in this meta-analysis were a heterogeneous group who were likely not only at risk for AD but also had impairments in multiple domains or had potential cerebrovascular dysfunction. The impact of these differences on GSH remains to be elucidated.

### Limitations

Substantial heterogeneity was observed between studies in brain and blood GSH in AD and MCI. There may be other sources of heterogeneity that could not be assessed systematically among the included studies. For instance, many AD studies in this meta-analysis did not report disease severity, limiting the ability to perform subgroup analyses. Other factors which involve a lack of information and potentially contribute to heterogeneity include concomitant illnesses and medications, both of which may affect antioxidant status. All studies were also cross-sectional in nature, which limits conclusions that can be drawn. There was also significant risk of bias in brain GSH measurements in MCI and blood GSH measurements in AD. The meta-analysis was also limited by the small number of studies in MCI and AD studies reporting GSH in the brain. Each brain region was considered as a sub-study, as each region of interest constitutes an individual MRI experiment, although this increases the *n* and thus decreases variance since a publication can appear more than once. To mitigate this effect, the results from left and right regions were averaged where bilateral measures were available.

## Conclusion

This meta-analysis found evidence to suggest decreased blood levels of GSH in AD and intracellular blood GSH in MCI compared to healthy controls. This analysis strengthens the increasing body of work identifying altered antioxidant responses as a potential contributor to cognitive impairment. This study also reveals the variety of assay techniques used to measure GSH in both brain and blood and highlights the need for a uniform measurement methodology. There is a wide range of MRS sequences available to measure in vivo brain GSH, and while the current studies in AD and MCI suggests that MEGA-PRESS is a good candidate for technique standardization, recent advances in MEGA-PRESS have also allowed for simultaneous measurements of pairs of compounds such as GSH/γ-aminobutyric acid and *N*-acetyl aspartate/*N*-acetyl aspartyl glutamate in one acquisition [96, 97].

Standardization of measurement techniques, reporting of important patient characteristics such as disease severity, onset, and duration, as well as concomitant illnesses and medications, and additional studies in MCI would allow for better characterization of early biomarkers changes in different stages of cognitive impairment. Indeed, recommendations to incorporate the use of imaging and fluid biomarkers as part of the diagnosis on a broader scale has been recommended by newer National Institute on Aging and the Alzheimer’s’ Association working groups [65, 98, 99] would help to characterize endogenous antioxidant changes in early stages of disease and offer insight into GSH’s potential as a therapeutic target.

## Supplementary Information

**Additional file 1:** **Supplemental table 1.** PRISMA Checklist - Altered central and blood glutathione in Alzheimer Disease and Mild Cognitive Impairment: a meta-analysis.

## Data Availability

The datasets used and/or analyzed in this current study are available from the corresponding author on reasonable request.

## Abbreviations

AD: Alzheimer disease
Aβ: Amyloid beta
CI: Confidence interval
DSM: Diagnostic and Statistical Manual of Mental Disorders
DTBN: 5,5′-Dithio-bis(2-nitrobenzoic acid)
GSH: Glutathione
HC: Healthy control
MCI: Mild cognitive impairment
MEGA-PRESS: Meshcher-Garwood Point-Resolved Spectroscopy
MMSE: Mini-Mental State Examination
MRS: Magnetic resonance spectroscopy
OPA: O-Phthalaldehyde
OS: Oxidative stress
PRESS: Point resolved spectroscopy
SMD: Standardized mean difference
STEAM: STimulated Echo Acquisition Mode
